# Chemo-Free Treatment Using Anti-PD-1 Antibodies with Lenvatinib in Unresectable Gallbladder Cancer: PD-L1 May Be a Potential Biomarker for a Better Outcome

**DOI:** 10.3390/diagnostics13111833

**Published:** 2023-05-23

**Authors:** Tiantian Wu, Changsheng Pu, Xianjia Wu, Qiang Wang, Keming Zhang

**Affiliations:** Department of Hepatobiliary Surgery, Peking University International Hospital, Beijing 102206, China; puchangsheng@pkuih.edu.cn (C.P.); wangqiang1@pkuih.edu.cn (Q.W.)

**Keywords:** anti-PD-1 antibodies, lenvatinib, gallbladder cancer, chemo-free therapy

## Abstract

Background: Recently, anti-PD-1 antibodies plus lenvatinib has been administered in a series of solid tumors. Yet, the efficacy of chemo-free treatment of this combined therapy has seldom been reported in gallbladder cancer (GBC). The aim of our study was to initially evaluate the efficacy of the chemo-free treatment in unresectable GBCs. Methods: We retrospectively collected the clinical data of unresectable GBCs treated using chemo-free anti-PD-1 antibodies plus lenvatinib in our hospital from March 2019 to August 2022. The clinical responses were assessed, and PD-1 expression was evaluated. Results: Our study enrolled 52 patients, with the median progression-free survival being 7.0 months and the median overall survival being 12.0 months. The objective response rate was 46.2% and the disease control rate was 65.4%. The expression of PD-L1 in patients with objective response was significantly higher than those with progression of disease. Conclusions: For patients with unresectable GBC, when not eligible for systemic chemotherapy, chemo-free treatment using anti-PD-1 antibodies with lenvatinib may become a safe and rational choice. The expression of PD-L1 in tumor tissues may be correlated to the objective response, and thus is expected to be a predictor of efficacy, and further clinical studies are certainly needed.

## 1. Introduction

Gallbladder cancer (GBC) is one of the most common primary malignant tumors of the biliary tract, with a high degree of malignancy and an extremely poor prognosis, and of which the patients’ median OS is approximately only 4–7 months [1]. The worldwide occurrence of GBC is less than 2/100,000 individuals, but this has been recorded with extensive variance according to geographic distribution [2]. Various genetic and environmental factors are involved in the development of GBC, including chronic infection of gallbladder or/and environmental exposure to specific chemicals, heavy metals, and even many dietary factors. Obvious correlation has been reported between formation of GBC and female gender as well as certain geographical regions, which is due to the influence of various female hormones, cholesterol cycling, and salmonella infections [3,4]. The rate of female GBC is 2–3 times than that of male GBC worldwide [5,6]. It has been reported that the female hormone estrogen may increase cholesterol supersaturation in bile juice and contribute to gallstone-mediated GBC pathogenesis [7]. However, a different view has emerged previously to question the association of hormone receptor expression with GBC differentiation [8]. Other important risk factors for GBC include porcelain gallbladder, Mirizzi’s syndrome, and bile reflux, which also serve as predisposing factors [3]. Tobacco consumption, family history of gallstones, chemical exposure, high concentrations of secondary bile acids, and excessive fried food intake increase the risk for GBC [9,10], among which gallstones are regarded as a major risk factor for GBC in China [11].

The generally accepted staging system for GBC is the tumor–node–metastasis (TNM) classification. GBCs of stage I and II are often resectable. However, when diagnosed in China, the majority of GBC patients belongs to stage III or IV due to lack of typical clinical symptoms in the early stage. Local infiltration or distant metastases can usually be identified, leaving less opportunity for radical resection. In unresectable cases, patients have to rely on chemotherapy and radiotherapy as the major therapeutic strategy [12,13]. However clinical outcomes are usually poor because of drug resistance or intolerance to side effects [14]. Recently, immunotherapy and targeted therapy have achieved remarkable results in the treatment of a series of advanced malignant tumors [15,16,17,18]. Current studies have suggested the safety and efficacy of this combined therapy or sequential therapy of targeted therapy/immunotherapy with systemic chemotherapy for advanced gallbladder cancer [19,20,21]. However, for patients who cannot receive systemic chemotherapy, the selection and determination of drug regimens need to be explored. No relevant literature has been reported so far.

In our study, we focused on the clinical efficacy of chemo-free treatment using anti-PD-1 antibodies plus lenvatinib in unresectable GBC. Based on the evaluation of clinical responses, we believe that chemo-free treatment using anti-PD-1 antibodies combined with lenvatinib may be an effective and safe option for patients with unresectable GBC who were not suitable for or unwilling to undergo systemic chemotherapy.

## 2. Materials and Methods

### 2.1. Patients and Methods

Fifty-two patients with histologically confirmed stage III and IV unresectable gallbladder cancer were treated using anti-PD-1 antibodies combined with lenvatinib at Peking University International Hospital from March 2019 to August 2022. Clinical data were retrospectively collected, approved by the Ethics Committee of Peking University International Hospital on 1st March 2023 and conducted in accordance with the ethics standards of institutional and national research committees and the 1964 Helsinki Declaration and its later amendments or comparable ethics standards. As the study involved the retrospective analysis of clinical data, the requirement for written informed consent was waived.

### 2.2. Treatment

The treatment included anti-PD-1 antibody with a fixed dose of 200 mg (Camrelizumab) per 3 weeks. Lenvatinib was administered orally once a day at a dose of 8 mg concomitantly. During the treatment, we collected data including the date of treatment, drug dosage, radiological evaluation, laboratory data, and adverse events (AEs). All patients underwent pathological biopsy beforehand, immunohistochemical staining was performed to observe the expression of PD-L1 in tumor tissues, and the expression intensity of PD-L1 was reflected by the proportion of positive tumor cells (TPS).

### 2.3. Follow-Up and Clinical Outcome Assessment

Patients were followed-up in person every 8 to 12 weeks. Imaging examinations were performed to evaluate the efficacy, including computed tomography/magnetic resonance imaging (CT/MRI) and positron emission tomography/computed tomography (PET/CT) imaging. The clinical objective responses were measured and evaluated by professional radiologists of Peking University International Hospital based on the Response Evaluation Criteria in Solid Tumors version 1.1 (RECIST v1.1) and described as complete response (CR), partial response (PR), stable disease (SD), or progressive disease (PD) [22]. Progression-free survival (PFS), overall survival (OS), objective response rate (ORR), and disease control rate (DCR) were determined to evaluate clinical efficacy. AEs were assessed in accordance with the Common Terminology Criteria for Adverse Events, version 4.0. Once PD was identified or severe AE occurred, treatment using anti-PD-1 antibodies combined with lenvatinib was terminated and a multidisciplinary conference was carried out to decide further therapeutic strategy.

### 2.4. Statistical Analysis

Survival was estimated using the Kaplan–Meier method. PFS was defined as the period from the time of treatment using anti-PD-1 antibodies plus lenvatinib to disease progression or patient death due to any cause. OS stood for the period from the time of treatment with anti-PD-1 antibodies plus lenvatinib to patient death for any cause or the last day of the follow-up. The deadline of follow-up was February 2023. All statistical descriptive analyses were carried out using SPSS 25.0 (SPSS, Chicago, IL, USA) software. To compare the difference of PD-L1 expression between groups, ANOVA was performed. *p* < 0.05 was considered statistically significant.

## 3. Results

### 3.1. Baseline Characteristics

Fifty-two patients with gallbladder cancer were treated using anti-PD-1 antibodies plus lenvatinib as a first-line chemo-free therapy in our research. The demographics and baseline characteristics are summarized in Table 1. The median age was 69 years, and 61.5% of the patients were female. All patients (100.0%) belonged to the Chinese Han ethnic group and had an ECOG (Eastern Cooperative Oncology Group) performance status of 0–1, and thirty-nine (75%) patients had good liver function with a grade A Child–Pugh score. The histopathological subtype of gallbladder cancer was adenocarcinoma and 32 of 52 (61.5%) patients were poorly differentiated. The baseline level of carbohydrate antigen 19–9 (CA19-9) was 534.30 U/mL. All patients had metastatic lesions in their liver and lymph nodes. Pulmonary metastasis was examined in 12 patients (23.1%) and peritoneal metastasis was detected in 26 patients (50%). In accordance with the TNM stage, 20 patients were diagnosed with stage III gallbladder cancer and 32 patients with stage IV. None of them had undergone any surgery or systemic therapy before.

### 3.2. Clinical Outcome

**Figure 1:**
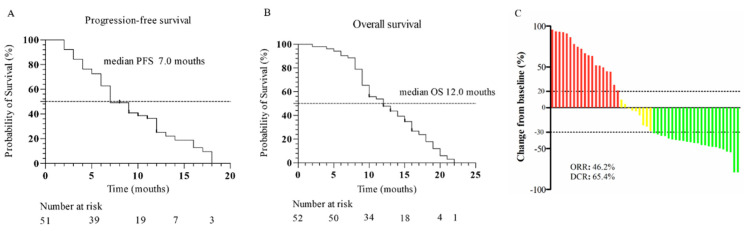
(**A**) Kaplan-Meier analysis of progression-free survival. (**B**) Kaplan-Meier analysis of ove-all survival. (**C**) Maximum percentage change in the sum of the diameters of the target lesions from baseline.

All participants in our cohort underwent imageological evaluations during follow-ups. PR was observed in 24 patients, SD in 10 patients, PD in 18 patients, and CR was not identified. The ORR was 46.2% (24/52) and the DCR was 65.4% (34/52) (Table 2). With a median follow-up of 12.0 (IQR: 7) months, the median PFS was 7.0 months (IQR: 8 months, Figure 1A) and the median OS was 12.0 months (IQR: 7 months, Figure 1B). Thirty-one patients (59.6%) exhibited a decrease in tumor size from baseline (Figure 1C). The survival outcome of the enrolled patients in the cohort were inquired and 10 patients found alive.

**Figure 2:**
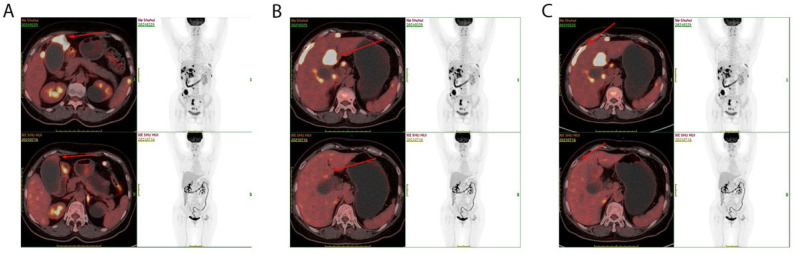
Positron emission tomography/computed tomography (PET/CT). (**A**) Glucose metabolism of the mass at the bottom of the gallbladder was decreased. (**B**) Glucose metabolism in the liver parenchyma around the gallbladder wall was decreased. (**C**) Glucose metabolism of multiple abdominal nodules surrounding liver capsule was decreased. The upper images (**A**–**C**) are the baseline images of PET/CT in February 2021, and the lower images (**A**–**C**) are the follow-up images of PET/CT in July 2021.

**Figure 3:**
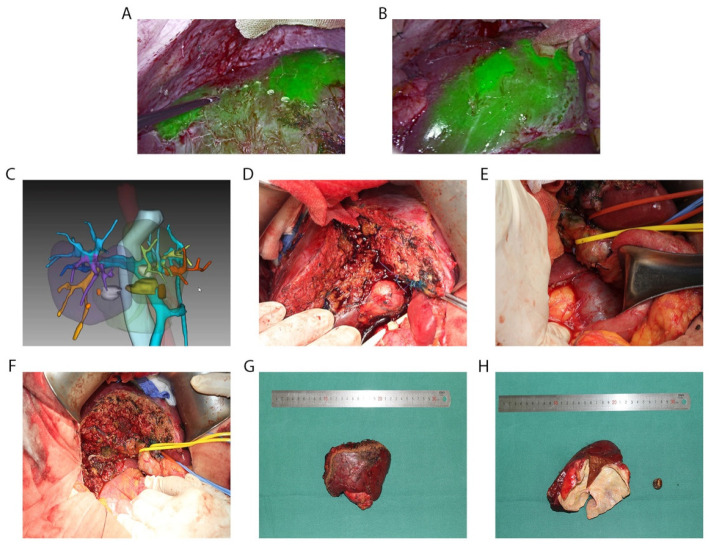
Radical resection of gallbladder cancer. (**A**) Fluorescent staining of segment 6 and 8 of liver (negative staining). (**B**) fluorescent staining of segment 4b of liver (positive staining). (**C**) Preoperative 3D reconstruction image of segment 4b and 5 of liver. (**D**) Gallbladder stones compressed the hepatic hilum. (**E**) Lymphatic dissection. (**F**) Cutting surface of liver. (**G**) Frontal view of the specimen. (**H**) Mucosal layer of gallbladder transformed to leather-like degeneration.

**Figure 4:**
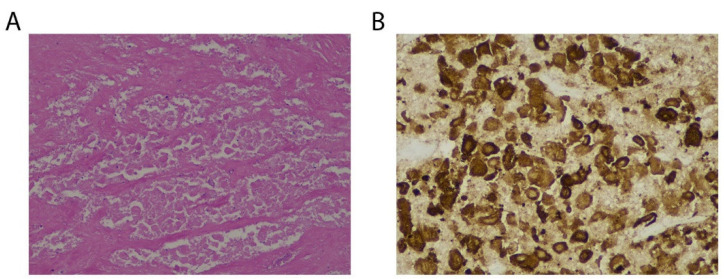
Postoperative pathological examination of bottom of gallbladder. (**A**) Hematoxylin and eosin, original magnification: 100×; (**B**) the positive staining of CK was observed. Original magnification: 200×. Pathological examination of the surgically resected specimen identified a pCR.

One patient was diagnosed as unresectable GBC using enhanced MRI and PET/CT, with intrahepatic, lymph node, and peritoneal metastases (Figure 2), and then was treated using anti-PD-1 antibodies combined with lenvatinib. After eight months of therapy, the metastatic lesions could not be found using an enhanced abdominal CT scan. Moreover, the glucose metabolism of the metastatic lesions was decreased to background level, revealed using PET/CT in October 2021. Thus, the stage of disease downgraded from IVb to IIIb and the disease was eligible for resection. A radical resection—including the gallbladder, segment 4b and segment 5 of the liver, and lymphatic dissection—was successfully performed on 3 November 2021 (Figure 3). Upon postoperative pathological examination, no obvious structure of adenocarcinoma was observed, and complete necrosis of GBC was detected in the bottom of the gallbladder (Figure 4), surrounding liver parenchyma, and resected lymph nodes. Pathological complete response (pCR) was identified. The patient restarted immunotherapy and targeted therapies two weeks after surgery and no recurrence was found during the follow-up.

### 3.3. Safety

Treatment using anti-PD-1 antibodies combined with lenvatinib was well tolerated among the patients. Apart from one patient’s discontinuance due to gastrointestinal hemorrhage, the main reason for discontinuation of treatment was disease progression. The median duration of the treatment was 7.0 (IQR: 6.0) months. No grade 5 AEs occurred and only one case of severe AE (gastrointestinal hemorrhage, grade 4) was reported. The majority of AEs included fatigue (43/52; 82.7%) and decreased appetite (39/52; 75%), which were classified as grade 1–2 in severity and were relieved using symptomatic treatment. Twenty-two patients experienced secondary hypertension (22/52; 42.3%) and 17 patients experienced diarrhea (17/52; 32.7%), both treated using symptomatic therapy. No unexpected side effects or treatment-related deaths were observed (Table 3). Four patients experienced reactive cutaneous capillary hyperplasia (RCCEP), which was relieved upon suspension of the anti-PD1 agent.

### 3.4. PD-L1 Expression

**Figure 5:**
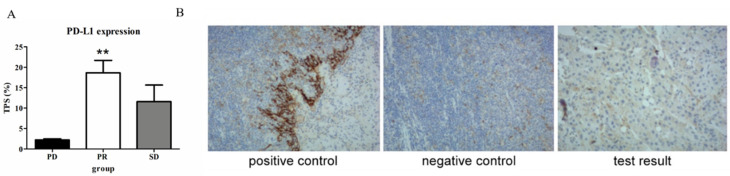
PD-L1 expression in gallbladder cancer. (**A**) The TPS of the objective response group (PR group) was higher than that of the PD group (18.66% vs. 2.21%, ** *p* < 0.001). (**B**) The immunohistochemistry of pathological specimen, the TPS of the patient who underwent surgery after successful conversion therapy was as high as 30% (100×).

Before the medication, all patients underwent tumor biopsy and PD-L1 immunohistochemical staining. The results demonstrated that differences existed in the expression of PD-L1. Patients were divided into three groups based on their objective response: PD group, SD group, and PR group. The proportion of PD-L1-positive tumor cells in patients of the PR group was 18.66% (95% CI:12.37%–24.94%), which was significantly higher compared to those in patients of the PD group (2.21%, 95% CI: 1.67%–2.75%, *p* < 0.001, Kruskal–Wallis test, Figure 5A). Meanwhile, the TPS of the patient who underwent surgery after successful conversion was as high as 30% (Figure 5B) in the PR group.

## 4. Discussion

Radical resection is probably the only curative choice for GBC to achieve long-term survival; however, in most GBC cases, patients are initially diagnosed with advanced local infiltration or distant metastases; only 30% of patients have the opportunity to undergo radical resection, while most patients need to undergo the comprehensive treatment, such as radiotherapy and chemotherapy [13]. The standard first-line chemotherapy for advanced GBC is gemcitabine and cisplatin (GC); however, ORR of GC is merely 30%, median overall survival (mOS) is only 11.7 months [14], and 5-year OS is less than 5%. Therefore, exploring new treatment options is crucial to improving patients’ survival. In addition, for patients with advanced GBC who are unable or unwilling to receive systemic chemotherapy, the selection and formulation of systemic medication has become an urgent clinical problem.

As a major breakthrough in immunotherapy, immune checkpoint inhibitors (ICIs) have been successfully applied in the treatment of lung cancer and melanoma [15]. These drugs relieve the immunosuppression of immune cells by blocking the PD-1/PD-L1 signaling pathway so that immune cells can regain antitumor function and kill tumor cells [23]. Therefore, ICIs could be used in the treatment of advanced GBC. Pembrolizumab is an immunoglobulin G4 (IgG4) monoclonal antibody PD-1 inhibitor. Kang et al. [16] studied 40 patients with unresectable or metastatic biliary tract cancer (BTC) who were treated with gemcitabine and cisplatin chemotherapy. By using pembrolizumab as a second-line (47.5%) or third-line (52.5%) treatment, median PFS (mPFS) and mOS were 1.5 (95% CI: 0.0–3.0) months and 4.3 (95% CI: 3.5–5.1) months, respectively. This suggests the potential efficacy of pembrolizumab in the treatment of advanced BTC. Another IgG4 monoclonal antibody PD-1 inhibitor, nivolumab, has also shown some efficacy in a clinical study, with an mPFS of 3.98 (95% CI: 2.33–5.98) months and mOS of 14.22 months (95% CI: 6.64–NA) [24]. However, some other studies have indicated that PD-1 or PD-L1 inhibitors alone are less effective in advanced biliary tract tumors [25]. Therefore, an approach using PD-1 or PD-L1 inhibitors combined with other medication may contribute to clinical efficacy in advanced GBC.

The vascular endothelial growth factor (VEGF)/vascular endothelial growth factor receptor (VEGFR) axis, which guides the formation of new blood vessels and new lymphatic vessels, is considered an important process in BTC tumorigenesis [26]. Two different studies have demonstrated that the density of microvessels and VEGF were critical independent factors for prognosis of GBC [27,28]. Recently, Xu et al. found that VEGF was significantly elevated in the serum of GBC patients, and suggested that VEGF promoted angiogenesis, cell proliferation, and invasion of GBC, while inhibiting cell apoptosis [29].

Therefore, exploring agents targeting VEGF/VEGFR has also become an option for GBC treatment. Lenvatinib is a recently developed anticancer agent that takes effect by inhibiting the expression of VEGFR1-3, fibroblast growth factor receptor (FGFR) 1-4, platelet-derived growth factor receptor α (PDGFRα), as well as the stem cell factor (SCF), and has been widely used in China for unresectable or metastatic hepatocellular carcinoma (HCC) [17]. Recent research [30] has shown that lenvatinib could inhibit cell growth and colony formation and induce apoptosis by increasing the expression of cleaved caspase-9, as well as regulate the cell cycle in the G0/G1 or S phase. In addition, lenvatinib may reduce cell migration and invasion by inhibiting the expression of matrix metalloproteinase (MMP)-2 and cell migration-inducing protein (CEMIP). Further studies in vivo have shown that lenvatinib could inhibit GBC cell growth through phospho-AKT (p -AKT), which is in line with the in vitro results [30]. As a monotherapy, lenvatinib was used in the second-line treatment of patients with unresectable BTC with a DCR of 88% and an mOS of 7.4 months [18].

Recent studies have shown that the anti-PD-1 antibody was able to enhance the effect of lenvatinib by altering the immune system [19], while lenvatinib affected the antitumor immune response by reducing the number of tumor-associated macrophages. When lenvatinib was used in combination with the anti-PD-1 antibody, the activation of the interferon (IFN) signaling pathway was enhanced and the anti-tumor effect was thereby improved [20]. Recently, Zuo et al. [21] reported a study on anti-PD-1 antibodies plus lenvatinib administered to patients with advanced GBC. Treatments before therapy using anti-PD-1 antibodies combined with lenvatinib included surgery, systemic chemotherapy, and radiotherapy. The mPFS was 5.0 months (95% CI: 4.1–8.0) and the mOS was 11.3 months (95% CI: 7.5–20.9). In general, no systematic clinical trials and data have ever been recorded on the chemo-free combination therapy of the PD-1 or PD-L1 inhibitor combined with lenvatinib, especially for unresectable gallbladder cancer.

Our research is the only specific study analyzing the chemo-free treatment outcomes of anti-PD-1 antibodies combining lenvatinib for unresectable gallbladder cancer patients to date. We focused on the evaluation of clinical efficacy and safety of anti-PD-1 antibodies combined with lenvatinib, as well as its potential contribution to surgical conversion. According to our findings, the enrolled patients achieved an ORR of 46.2%, a DCR of 65.4%, with an mPFS of 7.0 months (IQR: 8 months) and an mOS of 12.0 months (IQR: 7 months). The results demonstrated the feasibility and efficacy of anti-PD-1 antibodies combined with lenvatinib for stage III and IV unresectable gallbladder cancer patients unwilling to undergo or unsuitable for systemic chemotherapy. Compared with the research by Zuo et al., our study was based on a more homogeneous cohort—all the patients had not undergone any surgery, radiotherapy, chemotherapy before. Moreover, all patients were administered anti-PD-1 antibodies combined with lenvatinib as the first-line treatment, and we selected camrelizumab as the only anti-PD-1 antibody, which could minimize the heterogeneity caused by different anti-PD-1 agents. Our results may not be inferior to those in previous studies in terms of treatment outcomes. Of note, one successful surgical conversion was achieved, and a pCR was identified. To date, similar clinical efficacy has not been reported in the field of chemo-free systemic treatment. The therapeutic effect of anti-PD-1 antibodies combined with lenvatinib was encouraging and may indicate a promising chemo-free strategy for unresectable gallbladder cancer, with even the possibility of a cancer-free outcome.

All patients in our observation cohort underwent PD-L1 immunohistochemical staining. Studies have shown that PD-L1 expression is associated with the prognosis of ICC [31] and could be used as a predictor [32]. PD-L1 expression is an independent risk factor for poor prognosis in Western patients with gallbladder cancer [33]. However, under the condition of ICI treatment, the expression of PD-L1 may suggest a better curative effect. Studies have also shown that, in advanced BTC patients with positive PD-L1 expression, ORR and PFS are superior to PD-L1-negative patients after treatment with lenvatinib combined with a PD1 monoclonal antibody [21]. In our cohort, the TPS of the successful conversion case was 30%. The expression of PD-L1 tumor in patients with objective response was significantly higher than that in patients with disease progression. PD-L1 may thus be a promising indicator for predicting response, though this certainly requires further study.

For gallbladder cancer infiltrating the liver parenchyma, the scope of radical resection includes en bloc resection of S5 and S4b liver segments and the gallbladder, as well as lymph node dissection. Based on the concept of anatomical hepatectomy, we performed a three-dimensional reconstruction of the portal territory before surgery to determine the shape and volume of the parenchyma to remove and make sure whether the remnant volume was sufficient for life. In the operation, we used indocyanine green (ICG) staining and fluorescence navigation to guide liver parenchymal dissection. The outline of the resected parenchyma was almost the same as that of the preoperative 3D simulation. Shortly after the operation, the anti-PD-1 antibody and lenvatinib combination was administered again until the last day of follow-ups. Until now, there is still a lack of guidelines for the duration of immunotherapy, especially for patients with pathologically confirmed pCR. We think that pCR may have its limitations, and we thus maintained targeted therapy combined immunotherapy after surgery. Some studies have suggested that the minimal residual disease (MRD) is of great significance for evaluating the “true” complete response after conversion therapy, and we are actively preparing for relevant clinical studies.

Regarding the safety, although all patients reported adverse events during follow-ups, most AEs were limited to grade 1–2 severity and were curable. This suggests that the combination of the anti-PD-1 antibody with lenvatinib is a safe treatment and well tolerated for unresectable gallbladder cancer patients, especially in a chemo-free context.

There are some limitations to our study. Firstly, the sample size was limited, and it is necessary to enroll more patients in future research to overcome bias, and multicenter investigations are certainly of great importance. GBC is a rare malignant tumor and shows unusual geographic distribution worldwide. The rates of GBC among Mapuche people from Valdivia, Chile, South America are 12.3/100,000 for males and 27.3/100,000 for females [34]. Native Americans in New Mexico, USA, have an average annual rate of 8.9/100,000 [5]. Our cohort consisted of only the Chinese Han ethnic group. To our knowledge, there is still lack of detailed data considering the separate rates of GBC among different ethnic groups in China. Secondly, this study was a retrospective single-armed observation, with a low grade of evidence. Thus, for the sake of a higher grade, prospective randomized controlled trials may need to be carried out.

## 5. Conclusions

We presented the treatment results of 52 patients with stage III and IV unresectable gallbladder cancer who received a chemo-free medication of anti-PD-1 antibodies combining lenvatinib. Our findings lead to an observation that, in terms of chemo-free therapy for patients with late-stage unresectable gallbladder cancer, anti-PD-1 antibodies combined with lenvatinib could be a safe and effective medication, with possibility of surgical conversion. Similar successful cases of chemo-free therapy for late-stage gallbladder cancer have rarely been reported worldwide. Nonetheless, further prospective randomized controlled trials need to be conducted to confirm whether the medication of anti-PD-1 antibodies plus lenvatinib can serve as first-line drugs for unresectable gallbladder cancer. In addition, it is necessary to further explore the underlying mechanism of the synergistic effect of the anti-PD-1 agent combined with the tyrosine kinase inhibitor.

## Figures and Tables

**Table 1:** Baseline characteristics of the study population.

Parameters	Total (*n* = 52)
Race, *n* (%)	
Han	52 (100.0%)
Age, years (median, IQR)	69 (13)
<60 ≥60	14 (26.9%) 38 (73.1%)
Sex, *n* (%)	
Female Male	33 (63.5%) 19 (63.5%)
ECOG performance, *n* (%)	
0 1	29 (55.8%) 23 (44.2%)
Child–Pugh score, *n* (%)	
A B	39 (75.0%) 13 (25.0%)
CA19-9, U/mL (median, IQR)	534.3 (446.7–621.9)
<200 ≥200	7 (13.5%) 45 (86.5%)
Histology, *n* (%)	
Adenocarcinoma	52 (100.0%)
Differentiated histology, *n* (%)	
Poor Moderate Undifferentiated	32 (61.6%) 19 (36.5%) 1 (1.9%)
Site of metastases, *n* (%)	
Intrahepatic Lymph nodes Peritoneum Lung	52 (100.0%) 52 (100.0%) 26 (50.0%) 12 (23.1%)
TNM stage, *n* (%)	
IV III	32 (61.5%) 20 (38.5%)
Previous antitumor therapy, *n* (%)	
None	52 (100.0%)
Type of anti-PD-1 antibody, *n* (%)	
Camrelizumab	52 (100.0%)

ECOG—Eastern Cooperative Oncology Group; CA19-9—carbohydrate antigen 19-9; TNM—tumor–node–metastasis classification.

**Table 2:** Therapeutic efficacy of response and survival outcomes.

Therapeutic Response Assessment	Evaluable Patients (*n* = 52)
Confirmed objective response rate (ORR, *n*, %)	24 (46.2%)
Complete response (CR, *n*, %)	0
Partial response (PR, *n*, %)	24 (46.2%)
Stable disease (SD, *n*, %)	10 (19.2%)
Disease control rate (DCR, *n*, %)	34 (65.4%)
Progressive disease (PD, *n*, %)	18 (34.6%)

CR—complete response; PR—partial response; SD—stable disease; PD—progressive disease; ORR—objective response rate; DCR—disease control rate.

**Table 3:** Commonly observed adverse events.

Adverse Events (AEs)	Any Grade, *n* (%)	Grade 3, *n* (%)	Grade 4–5, *n* (%)
Fatigue	43 (82.7)	0	0
Hypertension	22 (42.3)	3 (5.8)	0
ALT or AST elevation	6 (11.5)	0	0
Decreased appetite	39 (75.0)	0	0
Abdominal pain	10 (19.2)	0	0
Vomiting	8 (15.4)	0	0
Diarrhea	19 (36.5)	1 (9.1)	0
Decreased weight	22 (42.3)	0	0
RCCEP	4 (7.7)	0	0
Gastrointestinal hemorrhage	1 (1.9)	0	1 (1.9)

ALT—alanine aminotransferase; AST—aspartate aminotransferase; RCCEP—reactive cutaneous capillary endothelial proliferation.

## Data Availability

The datasets generated during the current study are available from the corresponding author on reasonable request.
